# Evaluation of anti-A60 antigen IgG enzyme-linked immunosorbent assay for serodiagnosis of pulmonary tuberculosis

**DOI:** 10.4103/1817-1737.32229

**Published:** 2007

**Authors:** Zakeya Abdulbaqi Bukhary

**Affiliations:** *Section of Infectious Diseases, Department of Medicine, Taibah University, Madina, Saudi Arabia*

**Keywords:** A60 antigen, enzyme-linked immunosorbent assay, pulmonary tuberculosis, serodiagnosis

## Abstract

**BACKGROUND:**

Problems in the diagnosis of tuberculosis using smear and culture techniques have necessitated the exploration of the utility of serodiagnosis to support clinical suspicion of tuberculosis. Anti-A60 antigen IgG enzyme-linked immunosorbent assay (ELISA) was evaluated as a tool for the diagnosis of active pulmonary tuberculosis.

**MATERIALS AND METHODS:**

ELISA was used for the detection of immunoglobulin G (IgG) against A60 antigen in parallel with other familiar diagnostic methods in 70 patients with pulmonary tuberculosis (Group_I) along with 70 controls showing evidence of latent tuberculosis infection (Group II).

**RESULTS:**

ELISA results were positive in 63 (90%) patients in Group_I compared to three (4%) controls in Group_II. Group_I patients had significantly higher titers of IgG (mean = 750.79 ± 115.87 U/ml against the A60 antigen as compared to Group II controls (mean = 206.67 ± 20.81 U/ml (*P* < 0.0001). The overall sensitivity and specificity obtained using ELISA were 90 and 95.7% respectively in active pulmonary tuberculosis. Ziehl-Nielsen (Z-N) stains for acid-fast bacilli were positive in 60 (85.7%) patients. In 48 (68.6%) patients, *M. tuberculosis* grew on both Lowenstein-Jensen (L-J) medium and BACTEC MGIT 960 liquid medium with mean detection times of 45 and 8 days respectively. Tuberculin skin test was positive in 38 (54.3%) patients. Chest X-ray results were consistent with the diagnosis of pulmonary tuberculosis in 53 (75.7%) patients.

**CONCLUSION:**

Anti-A60 IgG ELISA results were significantly positive and associated with elevated antibody titers in pulmonary tuberculosis as compared to latent mycobacterium infection. The high diagnostic performance of the test makes it a useful, simple and rapid supporting tool to validate clinical suspicion of active pulmonary disease.

Serodiagnosis of mycobacterial infections has been the subject of various studies over the years as a fast and minimally invasive technique for the diagnosis and assessment of active disease.[1,2] Diagnosis of pulmonary tuberculosis (TB) depends mainly on the initial clinical suspicion and radiographic findings with subsequent microbiologic confirmation by direct smear microscopy and culture of sputum. Some of the disadvantages of traditional diagnostic techniques based on acid-fast bacilli (AFB) and direct smear microscopy are the lack of sensitivity and ofthe isolation and growth of the tuberculous bacillus in culture media are the length of time (sometimes growth takes several weeks).[3,4] Recent molecular biology techniques have made it possible to diagnose TB in a few hours, but they are expensive and not readily available in most hospitals in developing countries. In addition, these molecular biology techniques have low sensitivity particularly in smear- and culture-negative patients with pulmonary TB.[5,6] Hence, a rapid diagnostic method based on the detection of specific antibodies against *M. tuberculosis* using the enzyme-linked immunosorbent assay (ELISA) would be a simple, inexpensive and potentially practical tool for the diagnosis of pulmonary TB. ELISA was applied in 1976 to the serodiagnosis of TB using many semipurified, purified and immunodominant mycobacterial antigens.[3] Many antigens have been evaluated in order to develop a rapid TB diagnostic test, using culture filtrates as well as extracts from *M. tuberculosis* or *M. bovis*. Lately, purified protein derivatives (PPD) from *M. tuberculosis* and other semipurified antigens such as A60 from *M. bovis* strain bacilli Calmette-Guerin (BCG) have been used. These antigens showed variable specificities which were attributed to cross-reaction with similar antigens present in environmental mycobacteria or other microorganisms.[4] Many studies explored the use of the A60 antigen in the detection of TB antibodies from pulmonary and extrapulmonary sera with varying success (32.1–88.5%).[7] The A60 antigen is a thermostable complex present in the cytoplasm of exponentially growing mycobacteria. It is accumulated within the cell wall of stationary cells and released in the course of the active disease.[8] A60 is considered to represent a signal of T cell activation and was found to trigger both humoral and cellular immune responses. A60 has been reported to specifically activate T cells *in vitro*.[9,10] It has been observed that TB patients are usually positive for IgG antibodies rather than IgM directed against the A60 antigen.[11,12]

This study evaluated an anti-A60 antigen IgG ELISA method for serodiagnosis of pulmonary TB in 70 patients with active TB and 70 healthy controls with latent *Mycobacterium tuberculosis* infection.

## Materials and Methods

### Clinical investigation

#### Population and study design:

70 patients (Group I) who were consecutively diagnosed to have active pulmonary TB were studied after admission to the Chest hospital, Madina, Saudi Arabia between December 2004 to December 2005. The hospital has around 50 beds and facilities which only allow Z-N stain in addition to CXR. Culturing and ELISA were done at a reference laboratory in the same city. Samples were collected by nurses and patients' family members assisted in driving and transporting the specimens. In all patients, the following tests were done: full clinical examination, TST, CXR, Z-N stains for AFB and *Mycobacterium tuberculosis* cultures from sputum.

Seventy Saudi individuals having latent tuberculosis (Group II), who were visiting the hospital for healthcare or preemployment medical check-up and who had positive TST but normal CXR were included as controls. TST was graded according to the size of the induration: negative if <5 (0–4.9) mm, intermediate positive if 5–9.9 mm, positive if 10–15 mm and strongly positive if more than 15 mm or necrotizing after 48 hours. CXR findings were considered to be consistent with the diagnosis of active pulmonary TB if they showed cavitary lesions, right upper lobe infiltrate, fibronodular infiltrate, miliary shadowing or hilar lymphadenopathy in symptomatic patients. All Groups were HIV-negative. ḻnformed consent was not obtained as taking serum samples was considered a procedure of minimal risk in those who were already hospitalized or undergoing routine medical check-ups.

### Laboratory investigation

#### Microscopic examination of AFB:

Three successive morning sputum samples were collected from all patients. The sputum samples were subjected to decontamination, liquefaction and concentration procedures using N-acetyl-L-cysteine and 4% NaOH methods using BBL Mycoprep (specimen digestion/decontamination kit) from Becton Dickinson (Cokeysville, Maryland, USA). Decontaminated samples were used to prepare smears for Z-N staining. Slides were examined carefully as described by the W. H. O laboratory training program. They were graded according to the number of AFB per microscopic field at X 800 magnification into: +1 if 1–9 AFB are found in 100 fields, ++2 if 1–9 AFB are found per 10 fields, +++3 if 1–9 AFB are found per field and ++++4 if > 9 AFB per field. A smear was considered negative if AFB was ≤3 per 300 fields.

#### Culture:

Suspensions were inoculated into conventional (L-J) and BACTEC MGIT 960 media.

#### ELISA (TB IgG) test:

Seventy serum samples were obtained from patients in Group I before treatment and from controls in Group II. The test was performed to detect lgG antibodies directed against the A60 antigen using commercially available kits (Anda Biologicals, Strasbourg, France) according to the manufacturer's instructions. Serum dilution of 1:100 was used in the assay. Positive and negative reference sera were included in all runs along with test sera. For measuring lgG levels, the OD values of the test sera were extrapolated from a standard curve constructed by plotting the optical density (OD) values of different reference sera against their IgG concentrations. For the ELISA technique, diluted samples were incubated with the (anti A60 IgG)-sensitized wells of a microtitration plate for an hour at 37°C. After washing, the wells were filled with a solution containing peroxidase-conjugated anti-human IgG complex. After incubation for 30 minutes at 37°C, the wells were washed and incubated with an enzymatic substrate and H_2_O_2_. The anti-IgG peroxidase complex binds to the anti-A60 IgG antibodies in sera which have been captured by the sensitized wells of the microplate. This cascade of events ending in the H_2_O_2_-catalyzed enzymatic reaction with the substrate results in a color change from colorless to blue. The reaction was stopped with H_2_SO_4_ resulting in a color change from blue to yellow. The intensity of the yellow color which was proportional to the anti-A60 serum IgG level, was read in a photometer. In order to cope with the unavoidable daily variations observed with ELISA results, calibrators were included in the kit. A value of two IgG sero-units has been empirically attributed to the calibrator that gives an absorbance of 0.4-0.5 under optimal conditions. The cut-off points for a positive ELISA test were <150 U/ml for the control Group_II and ≥ 250 U/ml for Group_I patients with suspected pulmonary TB. The higher cut-off for a positive result in active TB *vs* latent TB increased test sensitivity in healthy controls compared to Group I who were active selected cases of TB.

### Statistical analysis

Values of IgG titers in sera from Groups I and II were compared using SPSS 10.0 for Windows. Means were compared using independent unpaired t-test.

## Results

**Figure 1:**
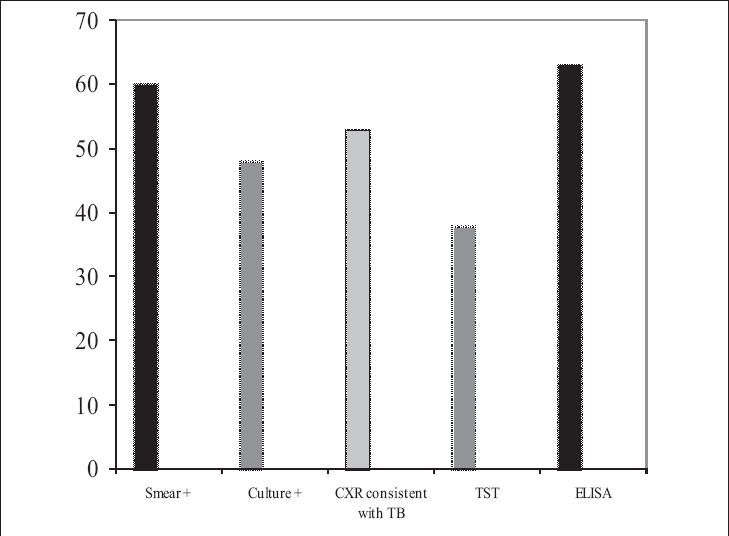
Number of patients testing positive by ELISA compared to other positive diagnostic tests (align properly) In Group I

140 sera samples obtained from 70 Group I patients and 70 Group II controls were studied using the ELISA described above. A summary of demographic and other features of the studied groups is provided in Table 1. Median age in Group I was 40 as compared to 37 years in Group II. Persistent cough was present in all patients (100%), expectoration in 53 patients (76%), hemoptysis in 49 patients (70%) and fever in 59 patients (84%). Twenty-two patients (31%) had a positive family history of TB. Five (7%) patients had associated extrapulmonary TB at the onset of pulmonary disease. Diabetes mellitus was the most frequently associated disease in ten cases (14.3%). The overall TST positive rate in Group I was 38/70 (54.29%) (54.3%) but was graded as positive in 7 (10%), strongly positive in 31 (44.3%) and negative in 32 patients (45.7%). Z-N stain for AFB was negative in ten (14.3%) patients and positive in 60 (85.7%). Smear was +1 in 23 patients, ++2 in 18, +++3 in 15 and ++++4 in six patients. Culture technique results were as follows: forty-eight patients (68.6%) showed positive growth indicative of *M. tuberculosis* in both conventional L.J. medium (mean detection time = 45 days) and BACTEC MGIT 960 (mean detection time of 8 days). The positive rates of all tests in Group_I is shown in Figure 1. A summary of the positive rates obtained with ELISA among the other conventional methods used for diagnosing pulmonary TB including direct Z-N stain, culture method and CXR has been presented in Table 2.

**Table 1:** Demographic and radiographic findings of studied population

Clinical data	Group I	Group II
Median age, years	41	37
(Range)	(17–57)	(20–50)
Male no. (%)	51 (73)	44 (63)
Female no. (%)	19 (27)	26 (37)
Country of origin		
Saudi Arabia	70 (100%)	70 (100%)
CXR		
Normal	0 (0%)	All normal
cavitary	35 (50%)	
Diffuse infiltrate	14 (20%)	
Miliary	4 (5.7%)	
Not consistent with active	17 (24.3%)	
disease		
Total	70 (100%)	
Associated diseases		
DM	10 (14.3%)	None
ESRD, HD	1 (1.4%)	
CLD	1 (1.4%)	

DM = Diabetes mellitus, ESRD = End-stage renal disease, HD = Hemodialysis, CLD = Chronic liver disease.

**Table 2:** Positive rates of enzyme-linked immunosorbent assay *vs* different diagnostic methods in Group I

Active pulmonary TB	Positive diagnostic test rate (no.)	Positive rate of ELISA no. (%)
Smear-positive	60	63 (90) (pg 5)
Smear-negative	10	3 (30)
Culture-positive	48	48 (100)
Culture-negative	22	15 (68.2)
CXR consistent with TB	53	53 (100)
CXR not consistent with TB	17	10 (58.9)
		3 (4.7)
		48 (68.6)
		15 (21.4)
		53 (75.5)
		10 (14.3)

TB - Tuberculosis, ELISA - Enzyme-linked immunosorbent assay

### ELISA:

Group I had a higher number of patients with positive ELISA results-63 (90%) compared to 3 (4%) in Group_II. IgG antibodies against the A60 antigen showed high titers in Group_I between 500-950 U/ml with a significant mean titer of 750.79 ± 115.87 U/ml as compared to positive titers in Group_II between 190–230 U/ml with a mean titer of 206.67 ± 20.81 U/ml, *P* value <0.0001.

## Discussion

ELISA as a test for diagnosis of TB still presents substantial variability in sensitivities (16-100%) and specificities (71-100%) and hence has not acquired a defined role in clinical practice.[13,14] In this study, a specificity of 95.7% for the serodiagnosis of TB maximizes the effectiveness of the test. This high specificity is considered to be an additional benefit to being rapid and inexpensive (it costs 2.93 US dollars (11 SR) and takes 2 hours to perform). The assay for anti-A60 IgG alone appeared to be adequate for detecting the immune response to *M. tuberculosis*.[15] All Group I patients had clinical successful response to anti-TB therapy. The positive predictive value (PPV) of ELISA method to access response to treatment. The antibody response was higher than expected compared to previous reports, probably because of long-term heavy exposure to bacilli in our geographical region in which TB is quite prevalent (2-14%).[16] In general, test sensitivity depends on both the investigator and the surveyed participants.[17,18] The sensitivity for detecting antibodies to the A60 antigen in patients with smear-positive pulmonary TB was reportedly high (83-88%)[19,20] while it was lower in smear-negative pulmonary TB (66.3%).[15] In this study, AFB smear sensitivity is compromised because > 10^4^ bacilli/mlof sputum are required for reliable detection.[3] *M. tuberculosis* culture was negative in one third of patients, most probably due to low bacillus load, decontamination method used and technical facilities in transporting the samples from chest hospital to the reference laboratory. In a majority (68.2%) of the patients with negative culture findings, ELISA results were positive. The positive predictive value (PPV) of the ELISA method used was 95.2% when compared to the AFB-ZN stain [Table 3]. In nonimunocompromized adult individuals who present with cough, fever, hemoptysis and positive AFB-ZN stain, positive anti-A60 IgG ELISA results of titers <500 U/ml strengthens the diagnosis for *M. tuberculosis* (MTB) infection. Conversely, negative ELISA results of titers < 250 U/ml may exclude a diagnosis of MTB infection and clinical suspicion would require further confirmation using PCR and culture techniques. In the presence of a direct relationship between positive ELISA results and elevated titers in active disease, the test may be of use as a marker during follow-up to assess response to treatment. It is well known that TST is more likely to be negative in the active phase of the disease due to suppressed T cell response. Furthermore, it is not useful in subjects with previous history of active TB or BCG vaccination.[21,22] Radiological features of CXR in pulmonary TB can mimic community-acquired pneumonia and are frequently atypical in patients with diabetes mellitus.[23–26] A positive anti-A60 IgG test in the presence of an abnormal CXR can help make an accurate clinical diagnosis of TB.[27]

**Table 3:** Diagnostic potential of enzyme-linked immunosorbent assay as compared to other methods

Anti-A60 IgG ELISA in Group I	Sensitivity %	Specificity %	*PPV	*NPV
∞ELISA compared to AFB Z-N stain	100	70	95.2	100
ELISA compared to culture	100	31.8	76.2	100

PPV: Positive predictive value, NPV: Negative predictive value

* Calculations not shown, can be released if requested.

∞ All positive tests had titers between 500–950 U/ml and negative tests had titers < 250 (190–230) U/ml

The major limitation of this study was the highly selective sampling in cases with suspected active pulmonary TB. This sampling may explain the high sensitivity rates in this study compared to those found in literature. Nevertheless, this selection gave us the advantage to prove the validity of the ELISA compared to other methods to diagnose active pulmonary TB. This body of work could be an initiative to implement a rapid serodiagnostic method for TB in developing countries and laboratories to avoid the cost of investing in sophisticated and expensive technology.

## Conclusion

Anti-A60 IgG ELISA results were significantly positive and associated with elevated antibody titers in active pulmonary disease compared to latent mycobacterium infection. The high diagnostic performance of the test makes it a useful, simple and rapid tool to validate clinical suspicion of active pulmonary disease.

### Future recommendations

The anti-A60 IgG ELISA should be evaluated in pulmonary and extrapulmonary TB and in children with BCGitis.
